# Lithium scale-making and extractivist counter-futurities in Bolivia

**DOI:** 10.1177/0308275X241269582

**Published:** 2024-09-15

**Authors:** Mark Goodale

**Affiliations:** 27213 University of Lausanne, Switzerland

**Keywords:** Bolivia, energy transition, extractivism, lithium, resource imaginaries, scale-making

## Abstract

This article uses the ethnography of the *prelives* of lithium industrialization in Bolivia to contribute to wider debates – in anthropology and beyond – about the essentially contested nature of the green energy transition. Based on research conducted between 2019 and 2023, the article examines the topographies of production and sociopolitical mobilization that are entangled with Bolivia’s state-controlled lithium project but which resist the various pressures to reorient social and productive worlds around arguably the most important ‘critical’ mineral for climate policy-making. The article develops a theoretical framework for understanding these localized counter-futurities, one in which the image of scale-making takes on both vertical and horizontal dimensions. An anthropology of energy, climate justice, and resource imaginaries that is critically attuned to these inter-scalar frictions is one that must also be able to project itself through the kaleidoscope of competing energy narratives as a form of both demystification and ethnographic truth-telling.

In August 2022, toward the end of the long, cold, and dry Bolivian winter, I stood with Alberto Colque Copa in the middle of a stone enclosure at a location called in Quechua ‘Hirucancha’, named after the area’s pervasive altiplano grass, *Hiru hichu* (*Festuca orthophylla*, known in Spanish as *Paja brava*). Colque had taken me to the Hirucancha to show me what I wanted to see, but also to show me what I needed to see. The stone enclosure was adjacent to a wood observation platform, or ‘mirador’. From this platform, one looks down from about 4,300 metres onto a mind-bendingly vast hole in the earth. This is Mina San Cristóbal, one the largest open-pit mines in the world, from which the Japanese company Sumitomo extracts silver, zinc, and lead, ships the minerals on a private railway to the Chilean port of Mejillones, and then transports them to various global commodities markets.

The mine was what I had come to see. Although the materialities of extractivism take many different forms, a massive open-pit mine like San Cristóbal perhaps best reveals – literally and figuratively – the horrific impacts of mineral extraction, the environmental violence, the sheer rapaciousness of resource capitalism. Even though the historic icon of plunder, Potosí’s Cerro Rico, or ‘Rich Mountain’, is only about 200 km from San Cristóbal mine, its dreadful ‘open veins’, as Eduardo Galeano (1971) described them, are hidden deep within the mountain, not truly open like the veins of San Cristóbal. The wooden platform at Hirucancha was built by the mining company itself, which assumed that the sight of such an immense extractivist cavity would be a point of pride for the residents of the surrounding communities and perhaps an object of touristic fascination for the few visitors who managed to find their way to the remote outpost (Figure 1).

**Figure 1.**
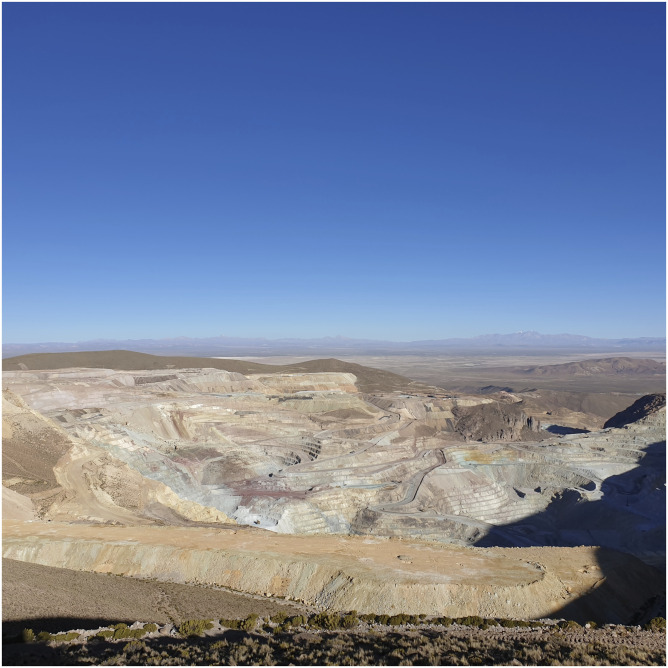
The view into the vast San Cristóbal mine. Source: Photo by Mark Goodale.

Looking down into the mine, I could just make out what looked like tiny trucks moving at many different levels; as I would later learn, these are among the largest trucks in the world, specially manufactured to transport hundreds of tons of ore at a time from the galleries of the mine to the processing facilities below. As I would also come to learn, several tyres from these trucks – which stand about 4 metres (13 feet) tall and cost over $40,000 each – have been arranged to lean together like a big rubber statue to mark the entrance to the small nearby town of San Cristóbal, from which the mine takes its name.

But as I gazed into the mine, Colque pointed to one of the striated walls, which appeared from that distance to be marked by different dark hues. As he explained, that section of the mine was among the newest. Although it was particularly valuable, it also was especially toxic. In its rush to expand that new level, Colque explained, Sumitomo had been ignoring incidents of sickness among the miners, who were working in large teams in 24-hour shifts. Given that productivity at the mine had suffered during the worst months of the recent Covid-19 pandemic, the company was pushing its workers in different ways to try to make up for lost revenue.

Even though I had come to see San Cristóbal mine, to see with my own eyes the material and productive embodiment of extractivist paradox – a Japanese-controlled mine that is destroying the surrounding altiplano while earning up to $1 billion per year after paying less than 10% in taxes and royalties to a Bolivian government officially committed to ‘productive sovereignty’, the protection of Pachamama, and a decolonial political economy organized around the logic of ‘living well’ – Colque really wanted to show me something else. We walked back to the stone enclosure, which consisted of four waist-level stone walls and, in the middle, a large stone table. As he said, the stone enclosure had nothing to do with the vast open-pit mine, but its location at the same spot as the wooden platform was not a coincidence. Rather, that location, Hirucancha, was one of the highest accessible points for hundreds of square kilometres, a place from which distant volcanos could be seen, the brown altiplano stretches to the horizon, and from where, looking north, one can see the transition zone into the Salar de Uyuni, where the Bolivian state is engaged in a fraught struggle to industrialize the world’s largest reserves of lithium.

Yet even though Bolivia’s lithium project is linked to the surging demand for lithium-ion batteries, which is linked to the surging demand for electric vehicles, or EVs, which is linked to the so-called green energy transition, which is itself seen as a key pillar of climate change mitigation on a global scale, the stone enclosure of Hirucancha is associated with climate processes with more bounded implications. As Colque explained, Hirucancha is where the authorities from all the surrounding communities gather at different times of the year, most importantly during a *cabildo*, or communal meeting, during the time of *Todos Santos* in late October.

During this gathering, llama foetuses are burnt and other offerings are made for one specific reason: to ask Pachamama to stop the wind blowing from the west so that the humid tropical winds from the north and northeast can be allowed to bring the life-giving rain on which everything else depends, from quinoa and potato cultivation to food for the llama herds. Standing there with Colque on that cold, cloudless, and parched late-winter day, the bright blue skies were stunningly beautiful (to me). At the same time, the sparkling blue of the altiplano sky was also a deadly warning: without clouds, there is no rain, and without rain, there is no life.

But are these ambiguously conjoined ‘sociomaterialities’ (Winther, 2013) – the private open-pit mine of San Cristóbal, the state lithium project on the Salar, and the fragile climate-dependent local agro-pastoral economy – examples of the kinds of ‘anthropocenic particularities’ (Boyer, 2019) that anthropologists of energy and climate change, among others, are being called upon to privilege, or do they intersect along other lines? And if they do provide the kind of ‘interillumination of translocal and local epistemics’ (2019: 7) through which the ethnography of resource, energy transition, and climate change can – and perhaps must – make its primary contribution, what broader lessons are to be learned from attending to granular materialities that are also embedded in wider – indeed, much wider – scales of potentiality, production, and desire?

As an initial response to these questions, this article explores the ways in which the region of southwestern Bolivia in which the country’s all-important lithium project is unfolding is one marked by complicated topographies that seem to resist the pull of the universalizing frames that are so dominant in current climate change discourse, whether about the global climate ‘crisis’ and its consequences, or about the post-anthropocenic futures that might serve as ‘counterfactuals’ (Colebrook, 2017), but only for the relatively few.

The fact that these topographies of production and sociopolitical mobilization are entangled with Bolivia's state-controlled lithium project is critical for understanding the wider implications of the article’s more grounded claims and contributions. As will be seen in more detail later, within a wider process that I describe as ‘lithiumphilia’ (Goodale, forthcoming; see also Goodale, 2024), that is, the global desire to control access to a resource that might very well become more ‘critical’ than oil in geopolitical terms within the coming decades, the struggle to extract Bolivian lithium has become particularly urgent. But because most of Bolivia's lithium remains trapped within the elusive brine that flows beneath the Salar de Uyuni, the sociomaterial worlds in which the project to industrialize lithium is embedded are profoundly affected by this enduring condition of ‘not-yet-actualized’ (Bryant and Knight 2019).

These sociomaterial worlds, which were the locus of my ethnographic research between 2019 and 2023, do not yet show either the ‘possibility of life in capitalist ruins’ (Tsing, 2015), or trace the afterlives of ‘unrealized material infrastructures’ (Yarrow, 2017).^1^ Instead, as I examine in this article, they reveal something quite different, what might be called the ‘prelives’ of the green energy transition, the diverse and often divergent quotidian realities of people enmeshed in the utopian resource imaginaries that emerge in relation to green energy futurities. But, as will also be seen, the nature of these entanglements is itself ‘fluctuating, heterogeneous, and not infrequently contradictory’ (Boyer, 2019: 7), something that also has wider theoretical and methodological implications.

In examining these implications, the article uses the concept of ‘lithium scale-making’ as a way of connecting the ethnography of social and productive life in this critical region of southwestern Bolivia to problems of wider concern to scholars of energy, the Anthropocene, and the green transition.^2^ In addition to engaging with the wider literature that rightly views scale-making as *both* a political and intellectual project, the article also introduces a complementary conceptual framework, one that allows me to analyse sociomaterial relations in the Salar region as a process of contestation over what I describe as ‘extractivist counter-futurities’. In a sense, these two serve as the conceptual *y* and *x* axes for the article’s arguments. The first is a way to better understand how the granular materialities of Bolivia’s lithium project can be projected into wider – if not necessarily planetary – frames of broader interest, while the second allows us to appreciate the relational and horizontal dimensions, the ways in which the lithium project in Bolivia must take its place among various other local and regional extractivist projects and futurities, none of which can really be said to offer an ‘antidote to the Anthropocene’ (Howe, 2019: 2).

After an initial section in which these and related theoretical dimensions are developed in more detail, the article then turns to the ethnography of lithium scale-making and extractivist counter-futurities. In returning to the region around the Salar, including San Cristóbal, I show how the wider green energy transition is also, in important ways, unfolding through its negations, its refusals, and its para- and counter-futurities. The article then concludes with a short section that reflects more generally on what the ethnography of the prelives of lithium industrialization in Bolivia reveals about the possibilities for life – and anthropology – in relation to a future that’s not coming (Goodale, 2023).

## Intersecting scales of energy, extractivist desire, and anthropology

Anthropologists of fossil fuel economies, energy generation (e.g. electricity), and the green energy transition alike grapple with how to situate grounded studies of particular political economic processes, particular stories of production and consumption, and particular narratives of ecological transformation, within a number of relevant broader frameworks, each of which has a claim to invest the particular with wider import. For example, anthropologists have reluctantly embraced the notion of human-induced planetary-level change, despite the almost instinctive hesitation to concede to universalist theoretical frameworks. Yet as Boyer, Faubion, and Marcus (2015) have argued, the need to develop telescopic methodologies has become more urgent than ever, for ethical as much as for epistemological reasons. For many, the ‘Anthropocene’ has become the accepted framework that allows researchers to link the specific impacts revealed through situated analysis to broader ‘conversation[s] about our earthly condition’ (Hecht, 2018: 111).

Moreover, ‘for many of the Anthropocene’s most prominent proponents, the term offers a way of signalling human responsibility’ (Hecht, 2018: 111). Boyer (2019) makes this point even more forcefully, insisting that the planetary-level frame of climate crisis and other global legacies of human plunder not be allowed to erase the radical disparity in liability. As he puts it, ‘the hailing of humanity as a species unfortunately obscures the differential culpability for global warming and environmental toxification, ignoring the fact that Northern empire has perpetuated these and other global conditions of precarity for centuries with impunity’ (2019: 6).^3^

At the same time, Indigenous researchers have demanded a new accounting of the Anthropocene, one that views the epoch as beginning with the violent social, economic, and environmental transformations that were unleased with the onset of colonial dispossession (Davis and Todd, 2017). And, as the Potawatomi scholar-activist Kyle Powys Whyte (2017) has argued, debates around lived experiences amid climate crisis, and the consideration of strategies for responding to climate and other forms of environmental change, must reckon with the embodied histories and practices within Indigenous knowledges, which reflect centuries of disruption, displacement, and resilience.

From a different perspective, scholars like Bruno Latour (2015) have embraced the Anthropocene for a quite different reason; rather than signalling differential responsibility, the ‘hailing of humanity’ offers a framework for identifying allies across boundaries, since the capacity for ‘telling friends from foes’ is the first step toward imagining social organization on the kinds of scales that will be necessary for confronting anthropocenic change.

A second broad approach to reconciling the challenges of giving priority to the ‘centrifugal stories’ (Nixon, 2014) of life in conditions of anthropocenic transformation while also ‘keeping the planet and all of its humans in the same conceptual frame’ (Hecht, 2018: 135) is to treat these problems of orientation as ethical problems. As Mette High and Jessica Smith (2019) have argued, in the introduction to a journal special issue on ‘energy and ethics’, by making the ethics of energy transformation an object of anthropological knowledge, scholars should resist the temptation to too easily slip into environmental judgementalism, which has the effect of narrowing the scope of inquiry in an otherwise actually diffuse field of discourse and action. And, as Jamie Cross (2019) has shown, adopting such a nuanced understanding of the relationship between energy and ethics opens some surprising critical doors, including those onto practices of humanitarian energy technologies that would otherwise appear relatively unassailable.

A third response to the methodological and theoretical questions around framing relationality in times of energy transition and ecological collapse focuses on the ways in which anthropocenic tensions are projected as imaginaries, ideational constructions – and conflicts over these constructions – of past, present, and especially the future. In an innovative recent study, Abram et al. (2022) argue that the theories and methods of futures anthropology should be productively brought into dialogue with the anthropology of energy to create what they describe as a ‘critical and interventional futures-oriented energy anthropology’, an approach that considers how alternative energy imaginaries can take the form of emerging technologies aimed at disrupting anthropocenic violence. From a different angle, Sean Field (2023) has shown how the plural in energy *futures* should be taken quite seriously, since they remind us that the climate crisis encodes struggles over ‘how value is assigned to worldly things’. In her study of the US coal market, Jessica Smith (2019) has made a similar point, except that here the struggle over energy futures – what she describes as ‘contested ethics’ – takes place *within* an energy regime rather than between visions for various alternative energy sources.

All of these strategies for moving between the anthropocenic particularities rendered visible through ethnographic research and broader frames – both conceptual and ecological – make important contributions to wider debates about the contested status of resource imaginaries within energy transitions. However, instead of locating my study of Bolivia's lithium project around one or the other of these different theoretical approaches, I want to keep them in view in a different way, by positioning them as ‘scalar claims of historical actors and projects’ (Hecht, 2018: 111) that also include my own (ethnographic) narratives, my own scale-making about, in this case, lithium scale-making in Bolivia. In this sense, Bolivia’s lithium project (or projects) must be seen as itself a scalar project, one with its own distinct analytics and politics, which – as with all scalar projects – must remain in ‘productive tension’ (Hecht, 2018: 111).

In adopting a scalar approach as a kind of conceptual grammar for talking about, and variously adopting, other approaches to relationality in an era of energy transition – the planetary, the ethical, the ideational – I am also able to attend to what is apparently excluded; or, rather, it allows for these apparent exclusions to become part of the scalar narrative, something critical for understanding lithium scale-making in Bolivia. As Hecht explains, ‘scale is not just about size or granularity. It is also about categories: what they reveal or hide, the ways in which they do (or do not) nest. And it is about orientation: how we position ourselves, what we position ourselves against, and what comparisons such locations do (or do not) authorize’ (2018: 114).^4^

But if the analytics and politics of scale-making, including my own, constitute the conceptual *y* axis for the article, my second move is to introduce a second axis, one that is meant to show how lithium industrialization in Bolivia is also unfolding along the horizontal plane, one in which local categories of production, identity, and ideology coexist dynamically – and not only in ways that reinforce existing structures of socioeconomic inequality and ecological vulnerability. As will be seen, the unfolding industrialization of the Salar de Uyuni is taking place on a broader regional landscape of communities that organize around what I describe as ‘extractivist counter-futurities’, which are logics of extractive production and political economy that stand as, in some cases, radical alternatives to the state-controlled lithium project.

This doesn’t mean that these existing economies and visions of the future have been crafted locally in direct contrast with the lithium project. On the contrary, they represent well-developed alternatives whose divergence from state industrialization only comes into view from certain perspectives, both ethnographic and analytical. In many cases, given the still-emergent nature of Bolivia’s lithium ambitions, the everyday importance of economic worlds that have nothing to do with these ambitions is perfectly understandable. The agro-pastoralists and miners of southwestern Bolivia have seen many ambitious national projects – both economic and political – come and go over the decades, none of which can be said to have had a truly transformative impact in the everyday lives of people. Given that the only real transformative project involves a private transnational mining company and the forces of global extractive capitalism, it is not surprising that the stalled plans for state lithium industrialization are viewed by many in the region with a revealing combination of scepticism and indifference.

But to return to the theoretical division between the vertical and horizontal axes along which the lithium project in Bolivia must be understood, here the question is not how lithium industrialization in Bolivia is situated in relation to the broader, vertical frames of global energy transition and climate crisis, but how ‘centripetal force’ (Nixon, 2014) is imposed laterally across sites, logics, and communities bounded by the general outlines of shared histories. The lithium project, like all such new energy projects that rely on extraction and the organization of productive labour, is interconnected in complicated ways with local and regional political economies that, to a certain extent, set limits to how lithium in Bolivia will eventually get taken up into the global circulation of ‘critical’ minerals on which the energy transition depends. Although I will come back to this question in the conclusion to this article, here it is enough to simply signal that attention to *both* the vertical and horizontal dimensions offers a framework for charting a much more expansive range of relevant anthropological particularities, to give a more nuanced account of what might be thought of as the actually existing energy transition.

With these various theoretical and disciplinary stakes at hand, let me now turn to a selective ethnography of the vertical and horizontal dimensions of Bolivia’s lithium project, the ways in which lithium scale-making and extractivist counter-futurities offer equally necessary frames of reference through which to analyse the project and unpack its lived ramifications.

## Welcome to the lithium capital of Bolivia

**Figure 2.**
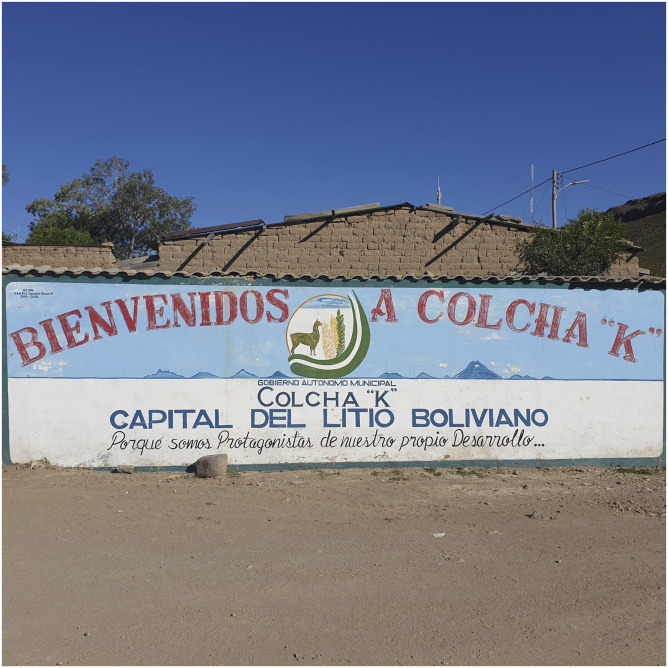
Mural at the entrance to the town of Colcha “K” near the Salar de Uyuni. Source: Photo by Mark Goodale.

At the entrance to the town of Colcha “K”, from which one can see the state lithium facilities on the Salar far in the distance, one is greeted by a large painted public mural. The mural is divided into two parts. The bottom half depicts the surface crust of the Salar itself, painted, as one might imagine, all in white, since the crust is composed mostly of halite, or rock salt, which, under the glare of high-altitude sunlight, becomes an ‘eerie, otherworldly sea of salt that will haunt your daydreams for years to come’, as one guidebook memorably described it. The top half of the mural depicts a wide blue sky and some of the local mountains, which include the volcano Tunupa, which has traditionally been conceived as a powerful female earth deity (Figure 2).

In the middle of the top half is the *escudo*, or crest, of Colcha “K”, a llama standing on the Salar with two quinoa plants. Stretching across the top half of the mural, in big, red, uppercase letters, a banner announces, ‘WELCOME TO COLCHA “K”’. And in a continuation of this bold welcome, the banner continues across the bottom half of the mural, ‘Lithium Capital of Bolivia’. At first sight, this is a curious assertion from the town, especially given that Colcha “K” plays no obvious role in the state-controlled lithium project, a situation made even clearer by the fact that the ‘subsurface resources’ of the Salar – meaning brine and the different minerals it contains, such as potassium, magnesium, but most importantly lithium – had been declared a ‘reserva fiscal’, or excluded production zone, long before the current Movement to Socialism (MAS) government decided to nationalize the industrialization of the Salar.^5^

But right under this striking act of political positioning, the banner continues onto a third line, which is less a declaration and more an explanation, one that seems to answer the doubts raised above. Written in cursive rather than block script, the line reads, ‘Because we are the protagonists of our own development …’ (with the ellipsis in the original). To understand what this means for Colcha “K”, its relation to the state-controlled lithium project, and, more broadly, the vertical dimensions of the green energy transition, something must first be said about the town’s scalarity. Colcha “K” is the capital of one of the most perplexing provinces in Bolivia: Nor Lípez. Nor Lípez is the largest of two provinces that were created from the historical region of ‘Los Lípez’ (the other is Sud Lípez).

People from this remote region of southwestern Bolivia, near the border with Chile and its equally remote Atacama region,^6^ have a strong identity as *Lipeños*, a fiercely independent and self-reliant ethnic group that was never fully incorporated into the Spanish colonial regime, particularly its forced labour system. In expressing this ongoing sense of Lipeño identity, people emphasize a number of characteristics and social values, including the capacity to endure in the face of difficult environmental conditions, complete economic autonomy, and a deep scepticism of both the departmental and national governments, including state control of key industries like mining.

At the same time, as the capital of Nor Lípez, the municipal government of Colcha “K” is responsible for managing millions of dollars a year in royalties and distributing these funds among 46 communities in the province.^7^ These relatively vast amounts are paid not by the Bolivian state, but by the Japanese owners of San Cristóbal mine, which is located in Nor Lípez.

I asked Cornelio Gonzalez, the charismatic 36-year-old president of the municipal council of Colcha “K”, why the town claimed to be the ‘lithium capital of Bolivia’ when it didn’t seem to have much to do with the project. Among other things, almost none of the almost 250 workers at the lithium plant, who are employed by the state lithium company Yacimientos de Litio Bolivianos (YLB) – from geologists to drivers to the cooks in the plant’s large mess hall – came from any of the communities in Nor Lípez, including Colcha “K”.

We [Lipeños] have always worked on the Salar, including for salt. Also, our quinoa fields go right down to the border. What we are saying is that Colcha “K” must have a voice in how the lithium project develops, especially compared to [the city of] Uyuni.

But why, I then asked, is Colcha “K” not mobilizing more formally to make demands on YLB? Why do so few people in the town itself talk about the lithium project?

Because people here have no confidence that the [national] government will ever manage to complete the project. YLB is a state company, just like COMIBOL [the state mining company]. COMIBOL mines produce almost nothing any more. In this area [Nor Lípez, but also southwestern Bolivia] there are three models. There is the model of the cooperative [mine], which produces some wealth for individual communities, but very little.^8^ There is the model of COMIBOL, where miners have low salaries and have to work with ancient equipment. And then there is the model of San Cristóbal.

What makes San Cristóbal so different?

The Japanese bring in the best technology in the world, they are not corrupt [like COMIBOL officials], they take care of their workers in case there is an accident, and the salaries, as you might know, are much higher than COMIBOL pays, much higher. Lipeños will always prefer the San Cristóbal model.

I pursued these questions about the multi-scalar dimensions of the lithium project further with Yamile Cruz. Cruz was the second half of the political power couple that included her husband Cornelio Gonzalez, the president of the municipal council of Colcha “K”. And, given her position, Cruz was arguably a more influential figure in regional politics and social organization than her husband. In 2022, Cruz had just been elected as one of the youngest, and the first female, leader of the Federación Regional Única de Trabajadores y Campesinos del Altiplano Sur (Regional Federation of Workers and Peasants of the Southern Altiplano), known as FRUTCAS. When I asked her about what it meant to be the first female leader of FRUTCAS, she told me, ‘I am more Bartolina than Mujeres Creando’.^9^

But what I really wanted to discuss with Cruz was FRUTCAS’s strategy in relation to the state lithium project. FRUTCAS, which is one of the historic *sindicatos campesinos*, or ‘peasant unions’ that was founded after the 1952 National Revolution, exercises sway over a vast stretch of southwestern Bolivia, which includes the communities of five separate provinces, including Nor Lípez. The traditional logo of FRUTCAS is a militant peasant in profile, raising his fist to the sky. As I mentioned to Cruz, I had noticed that the logo had been modified in the months after her election. The militant peasant remained, but underneath, below FRUTCAS and the names of the five associated provinces, something else had been added: ‘Region of Bolivian Lithium!’

I asked Cruz how FRUTCAS was planning to insert itself into what YLB was describing as the ‘entire productive chain’ of lithium even though the industrial production of lithium carbonate hadn’t yet started.^10^

We have two main objectives. First, we want YLB to recognize that the communities of Nor Lípez have a special relationship with the Salar. Any major economic activities here need to be developed through consultation. We are studying whether we can use provisions of the [2009] constitution to insist on free, prior and informed consent before any new facilities are built or deals are made [with foreign companies]. And second, we want to have a voice in the negotiations over a future lithium law. We believe that our special relationship with this region gives us the right to have a greater share of the royalties.

Knowing that FRUTCAS had opposed earlier attempts by the Bolivian government during the so-called neoliberal era of the 1980s and 1990s to open up the Salar to lithium extraction, I wondered why FRUTCAS was not opposing the project now. Had FRUTCAS considered the ongoing resistance by Indigenous (Atacameño-Likanantay) communities in Chile, which had seen their water supplies severely affected over the decades of industrial production of lithium, which is heavily water-dependent in a region that is among the driest in the world?^11^

We [in FRUTCAS] are obviously concerned about the impact on our communities, especially Río Grande [a community next to – and named after – the Salar’s most important source of water, the Río Grande]. But most of all, we are frustrated with the government. We have been participating in meetings, bringing our members together, for over ten years.

Are you frustrated that the government is investing so much money in the lithium project?

No, we are frustrated that the project is not more advanced. We have been waiting for it for a long time. The government tells us every year that the project will move forward and every year there are new delays. We will keep waiting for lithium, but we are getting tired of all the promises.

Yet if the communities and social movements of southwest Bolivia make a variety of ‘scalar claims’ (Hecht, 2018) in relation to the unfolding lithium project, claims that ramify both backward and forward across regional history and cultural identity, the lithium project must also be understood from a perspective of even starker contrast, that is, through adjacent sociomaterial worlds marked by negation, refusal, and the flourishing of para- and counter-futurities.

## Resisting lithiumphilia through extractivist counter-futurities

During the first period of research on Bolivia's lithium project in 2019, I was told repeatedly by a diverse range of people – from Salar tourist guides to municipal officials in Uyuni to research scientists in Potosí – that I should make sure to visit the town of San Cristóbal. People were somewhat vague in their reasons for this insistence: there was a lot of wealth in San Cristóbal; community members would probably have strong opinions about the lithium project; and the town was connected with a large mine that had nothing to do with the MAS government or COMIBOL, Bolivia’s state mining company. As it turned out, the status of San Cristóbal proved to be an important part of the wider story of lithium industrialization in Bolivia, in which the town has developed a distinct socioeconomic vision, one grounded in an alternative orientation to questions of extractivism. This is a contrasting vision of its social and productive future that is intentionally disconnected from the vague promise of royalties from the sale of lithium or, more generally, the state’s planned integration into a global energy transition.

Although I am undertaking a fuller critical analysis of my research in San Cristóbal elsewhere, the relevant context for this section can be summarized as follows.^12^ In the mid-1990s, a small group of exploration geologists conducted an initial survey in the hills above what people today call the ‘old’ San Cristóbal, which was a village of about 200 people located 20 km from the site of the current town of 2,500. The geologists had been hired by an American mining company, Apex Silver Mines, to locate new deposits of silver in South America. The team was led by an exploration geologist from Pennsylvania named Larry Buchanan, who had received a PhD in geology from the Colorado School of Mines in 1979. Among other things, Buchanan was well-known for having invented the ‘Buchanan Boiling Model’, a technique for extracting ore from hydrothermal fluids.^13^

Based on its initial findings, Apex Mining, now in collaboration with a Bolivian mining company called Mintec, expanded the pilot survey into a more systematic study of the region around the small village of San Cristóbal. What they discovered was a massive deposit of silver, zinc, and lead, which led Apex to immediately begin negotiations with the Bolivian government of the time, headed by the arch-neoliberal Gonzalo Sánchez de Lozada, which was in the midst of a widespread process of privatization of the country’s major industries, including mining. The complication, however, was the fact that the mineral deposit over a large area was found to be relatively close to the surface, something that usually points to the use of an open-pit mine. But what to do with the old village of San Cristóbal, its residents, its small colonial church, its ancient cemetery?

In 1998, after over a year of drawn-out negotiations between the mining company and the village leaders, a consensus was finally reached: the village would agree to its own destruction so that the open-pit mine could be built, but only if a long list of conditions was met.^14^ After further negotiations, which included a number of proposals and counter-proposals, the mining company agreed to these astonishing conditions. These included the transfer of the entire village to the new location, closer to the interstate highway that goes to the Chilean coast; the dismantling, removal, and rebuilding of both the church and cemetery (which included the work of architects and restoration experts); the construction of new houses for every family from the old village in the new town; the provision of necessary utilities, a school, roads, and all other infrastructure in the new town; jobs in Mina San Cristóbal for any community member who wanted one (although specific jobs were not guaranteed); and, perhaps most importantly, an agreement that the mining company would pay royalties to the new town, amounts that would later run into the millions of US dollars.^15^

By 2022, after over twenty years under this unprecedented *convenio madre*, or ‘mother agreement,’ the town of San Cristóbal had become a repository of unimaginable family wealth, in which community members – especially those who were ‘shareholders’ of the mother agreement from 1998 – had managed to accumulate the kinds of assets and financial profiles that are almost impossible to comprehend in relation to either the rest of small-town rural Bolivia *or* most of its urban working and middle classes. Between the international mining salaries, which can run as high as US$4,000 *per month* (the average public teacher salary in Bolivia is around US$500 per month), the payment of 20 years of annual royalties to the town from the mining company (which is now Sumitomo, which bought the rights to the mine from Apex Silver), and individual investment by families in things like second homes in Cochabamba and Tarija, the town of San Cristóbal represents an alternative universe partly of its own creation. This universe exists as a kind of political economic antipodes to the state lithium project, one built around an extractivist futurity that runs directly counter to the Bolivian government’s policy of national ‘productive sovereignty’ and post-neoliberal development.

But how do the beneficiaries of this unique ‘pacto de reciprocidad’ (Platt, 1982) imagine it in relation to the highly visible lithium process unfolding just down the highway? As Elsa Rivera Gonzales explained, community members in San Cristóbal were not interested in the state lithium project, in part because their relations with Sumitomo had shown them what it was like when an ‘international’ company was in charge of a major project, in this case one with which the town was entangled in a complex web of interdependence. Rivera Gonzales, who in January 2022 had just been elected as the town’s *corregidora*, one of its most important positions of authority, drew a sharp contrast with the mines in the wider region that were still controlled by COMIBOL, the Bolivian state mining company.

COMIBOL mines are completely different from ours [Mina San Cristóbal]. They are run by corrupt bureaucrats who have their jobs because they are connected to *oficialísmo* [the political powers-that-be]. The government doesn’t have the money to invest in equipment like international companies and of course the salaries are much lower. YLB [the state lithium company] will be just like COMIBOL so why would we want to collaborate with YLB?

Ronald Alí Yañez, a young ex-*corregidor* of San Cristóbal, and a member of one of town’s original ‘shareholder’ families, was even more forceful in his dismissal of the state lithium project. Alí, who was the leader of a group of ambitious community members that called itself the ‘Dreamers Club’, was working on a long-term plan for San Cristóbal, one that didn't involve either the local mine (which will be exhausted by 2050, according to company estimates) or the lithium project. This somewhat unlikely plan calls for San Cristóbal to reconstitute itself eventually as a ‘dry port’, something like an inland *entrepôt*, where both Bolivian and international companies would pay to store goods and materials – including ore from other mines – before and after their arrival at the Chilean trans-shipment ports 500 km to the southwest. I asked him why people in San Cristóbal were sceptical and even contemptuous of the state lithium project, given its importance to the national government.

Listen, we are only a little municipality, but we have the wealth of Dubai. This gives us the opportunity to make our own future, to follow our own vision. The current lithium model [state-controlled] is completely wrong. We don't want any state interference in our local or regional industries.

I then asked Alí if San Cristóbal maintained good, bad, or indifferent relations with either the national or departmental MAS government, especially given that a number of important regional leaders in Nor Lípez were members of the party. What did he think, for example, about the widely discussed possibility that the government might move to complete its policy of productive sovereignty by nationalizing the country’s largest mines, including Mina San Cristóbal? Alí scoffed at the idea:

The only thing that would cause us to mobilize would be if the MAS tried to nationalize the mine. Then there would be a real uprising [levantamiento].

## Conclusion: Lithium scale-making and extractivist counter-futurities within and against planetary frames

To conclude, let me return to the underlying theoretical stakes before briefly reflecting on what the ethnography of the *prelives* of lithium industrialization in Bolivia has to contribute to wider debates – in anthropology and beyond – about the essentially contested nature of the green energy transition. As Gabrielle Hecht has argued, the key challenge for critical energy scholars is to devise conceptual frameworks that coherently keep ‘the planet and all of its humans’ (2018: 135) in constant focus, even if only for strategic reasons, while at the same time making space for divergences at all scales, the kinds of divergences that remind us that lived experience in and of the Anthropocene will never mirror the global scales of the problems themselves: climate crisis, ecological breakdown, global warming, and so on.

At the same time, if scale-making as a device has proven to be useful in negotiating these ethnographic and theoretical challenges, we must be careful to keep the sense of scale itself open to interpretation and the possibility of being ‘recrafted’ (Winchell, 2022). As Tsing (2012) has put it, when we insist on a narrow approach to scale as both method and form of world-making, especially the idea of what she calls ‘precision-nested scales’, we do violence to the ‘diverse and dynamic … living world[s]’ that come to flourish – or at least survive – in the ‘mounting pile of ruins that scalability leaves behind’ (Tsing, 2012: 505).

My own response to these dilemmas has been to divide the conceptual framework through which to organize the diffuse and often surprising ethnography of Bolivia’s lithium project into two axes. The *y*, or vertical axis, highlights the distinctly *non*-precision-nested scales that interconnect local imaginaries of lithium extraction (whether realized through production or not) to national energy policy-making and scales beyond, including the global green energy transition itself. The nature of these interconnections is partly explained by the increasingly urgent quest to gain access to the world’s largest reserves of lithium. But it is also explained by pre-existing logics of resource dependency and the histories of (neo-)extractivism that have enmeshed Bolivia (in its different political forms) for centuries.

And my second manoeuvre has been to intersect this more well-established analytics and politics of scale-making, including my own, with a horizontal axis, one that extends the critical gaze outward from the more obvious sites of lithium extraction and energy policy to show how what might be described as the actually existing green energy transition is deeply intertwined with contested counter- and para-visions of production, governance, and community autonomy. As the ethnographic illustrations revealed, even something as critically important to the broader global energy transition as lithium industrialization in Bolivia is buffeted by both centrifugal and centripetal forces (Nixon, 2014), yet forces that cannot be assimilated easily to the prevailing grand narratives, whether ecological or political economic. An anthropology that is critically attuned to these fundamental tensions and *décalages* – in the untranslatable sense of ‘lack of correspondence’ – is one that must also be able to project itself through the kaleidoscope of competing energy futurities as a form of both demystification and ethnographic truth-telling.

## Notes

1. Of course, this is not to say that lithium extraction in other countries is not associated with sociomaterial or ecological afterlives; indeed, quite the contrary, which is part of what makes Bolivia’s unfolding lithium project so revealing. For a study of ‘exhausted ecologies’ in the long-standing zone of lithium extraction in Chile, see Blair et al. (2023). Thea Riofrancos’s work (e.g. 2023) has explored, among other things, the impact of lithium mining in the global North, where extractivist desire is closely tied to national security and the projection of imperial power.

2. It should be noted that the concept of the ‘green transition’ has been rightly contested by scholars. For an example of these debates, see Dunlap (2023).

3. The deep unease with the elisions and misdirections permitted by the centring of our species within *Anthro*pocene has led scholars such as Jason Moore (2015) to both privilege the question of blame (rather than effects) and point the analytical finger directly at the source, the Industrial Revolution and the rise of the corresponding ‘Capitalocene’. Despite these efforts at conceptual distinction, Donna Haraway (2015), no stranger to theoretical experimentation herself, has poked fun at the proliferation of competing proposals for global epochs. See also the recent series of essays on ecologies, nature–society relations, and mobilities organized around the concept of the Plantationocene (Barua et al., 2023). Finally, the Chilean scholars Manuel Tironi and Beltrán Undurraga (2023) have argued for an approach to the Anthropocene that reverses the angle of regard: in addition to understanding the social dimensions of planetary change, they insist that we must also ‘geologize’ our social worlds.

4. For a powerful study of how scales of environmental science and land management have been deployed historically as a tool of colonial violence, see Liboiron (2021).

5. The question of the legal status of the Salar's brine is too complicated – and convoluted – to discuss in any detail here (but see Goodale, forthcoming). However, it is important to note that the intention behind the imposition of this unusual category (‘reserva fiscal’) was twofold. First, to ensure that a succession of Bolivian governments would have the right to make decisions about the Salar’s brine without having to negotiate with departmental officials. And, second, to reduce the likelihood that the many smaller political units that have geographical and/or cultural ties to the Salar – cantons, municipalities, provinces – would become locked in a never-ending struggle over future royalties from the sale of lithium carbonate.

6. The Atacama region, which now includes many of Chile’s main lithium production facilities, used to be part of Bolivia, until the War of the Pacific (1879–84), when the territory was occupied and then annexed by Chile as part of its victorious military campaign against Bolivia and Peru.

7. The jurisdictional alignments here are a bit more complicated than presented, in which ‘municipalities’ are not the same as towns, but *municipios* refer instead to what used to be sections of provinces before a national reform of political boundaries and positions (for example, heads of departments used to be prefects and now they are governors).

8. The geographer Andrea Marston has conducted landmark research on cooperative mining in Bolivia, a form of resource extraction that has its own vertical and horizontal dimensions. See, for example, Marston (2019, 2024).

9. Knowing of my long anthropological engagement with Bolivia, Cruz was able to use this categorical shorthand to position herself as a woman working in an elected office of significant social and political responsibility, at least at a regional level. The ‘Bartolinas’ are the universally used name for one of the most powerful social movements in Bolivia and one of the five national movements that committed to a ‘Pact of Unity’ in support of Evo Morales and the MAS government’s *proceso de cambio.* As a women’s movement comprised largely of Indigenous women, the Bartolinas combine political radicalism in support of Indigenous communities and issues with a kind of social conservatism around marriage, family, and sexuality. Most Bartolinas would not use the word ‘feminist’ to describe themselves, as I learned from Patricia Choque, the head of the Bartolinas during the first Morales administration (Goodale, 2019). Mujeres Creando, however, represents a very different strand of female organizing. It is an anarchist and explicitly feminist collective based in La Paz. The collective is known for its direct action street performances, the use of political graffiti (with a signature tag), and its ‘permanent rebellion’ on behalf of members of Bolivia’s LGBTQ communities.

10. For example, on one of the walls of YLB headquarters in La Paz, a large poster shows the industrial facilities at the Salar at sunset, beaming with high-voltage electricity, along with the phrase, ‘Bolivia is the only country in Latin America that has taken up the industrialization of lithium across its entire productive chain.’

11. Yet even among the Indigenous communities of Chile, the strategies adopted in relation to lithium production are more complicated than a simple (and expected) story of resistance to extractivism. See, for example, Sally Babidge’s forthcoming study of the politics of water in a region of the Atacama with both copper mines and lithium production facilities. For an account of community mobilization against hydrocarbon development in another region of Bolivia, see Anthias 2018. See also Gustafson 2020.

12. See also the remarkable, if idiosyncratic, history of San Cristóbal published by the Bolivian diplomat, journalist, and historian Mariano Baptista Gumucio (2015).

13. As the citation for his distinguished achievement medal from ‘Mines’ put it, the use of Buchanan’s model ‘has since helped guide geologists to billions of dollars’ worth of silver in thermally active regions’ (Colorado School of Mines, 2013).

14. This stage in the process took place under the democratic government of Hugo Banzer Suárez, the former military dictator who had ruled Bolivia between 1971 and 1978.

15. The payments to San Cristóbal and other investments, including the creation of a local development agency called Fundación San Cristóbal, were in addition to the royalties paid to authorities in Colcha “K” to be distributed among the communities of Nor Lípez province.
